# Misdiagnosis of bilateral tubal pregnancy: a case report

**DOI:** 10.1186/1752-1947-8-342

**Published:** 2014-10-14

**Authors:** Weifeng Li, Gang Wang, Tiecheng Lin, Wenwen Sun

**Affiliations:** 1 Department of Obstetrics and Gynecology, The First People’s Hospital of Foshan, No. 81, North Lingnan Street, Chancheng District, Foshan, Guangdong 528000, China

**Keywords:** Bilateral tubal pregnancy, Laparoscopy, Misdiagnosis

## Abstract

**Introduction:**

The incidence of bilateral tubal pregnancy is rising due to the increase of pelvic inflammatory disease and assisted reproductive techniques. Because the clinical manifestations of bilateral tubal pregnancy are not specific, we often ignore inspection of the other fallopian tube when focusing on the lesions, which may cause misdiagnosis.

**Case presentation:**

A 33-year-old Chinese woman presented with vaginal bleeding after menopause and with an abnormality found by transvaginal ultrasound scan for which she underwent laparoscopy and salpingectomy. Unfortunately, she had to undergo a repetitive laparoscopic salpingotomy for the other tubal pregnancy due to misdiagnosis of her bilateral tubal pregnancy.

**Conclusions:**

The incidence of unusual presentations of ectopic pregnancies has risen. Surgeons should always keep in mind the possibility of bilateral tubal pregnancy. An attentive examination of the pelvis, especially the two fallopian tubes, is necessary to avoid missing bilateral tubal pregnancy.

## Introduction

Bilateral tubal pregnancy (BTP) is a very rare type of ectopic pregnancy. The incidence of bilateral tubal pregnancy is 1 in 725 to 1580 ectopic pregnancies and 1 out of 200,000 pregnancies [1]. The occurrence has tripled in the last decades with most cases being associated with previous assisted reproduction techniques (ART), with the use of intrauterine contraceptive devices (IUD), with pelvic inflammatory disease (PID), history of ectopic pregnancy or following tubal surgery [2,3]. We report a misdiagnosed case of BTP that occurred in The First People’s Hospital of Foshan, China on 12 December 2013.

## Case presentation

**Figure 1:**
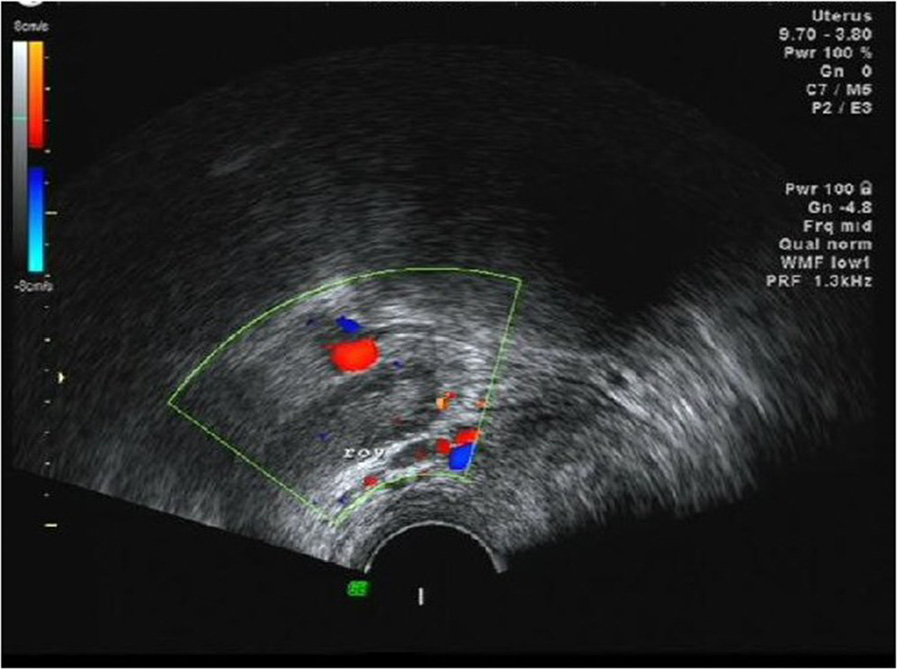
An ultrasound scan (on 13 December 2013) showed a right adnexal mass and nothing abnormal was detected in the left attachment area.

**Figure 2:**
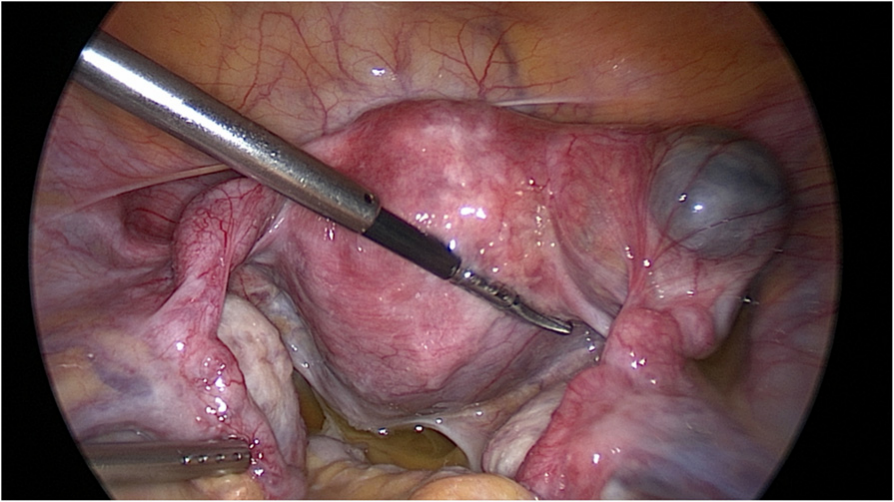
During the first surgery, a 40×50mm nearly ruptured right interstitial tubal pregnancy and a slightly thickened left tube were found.

**Figure 3:**
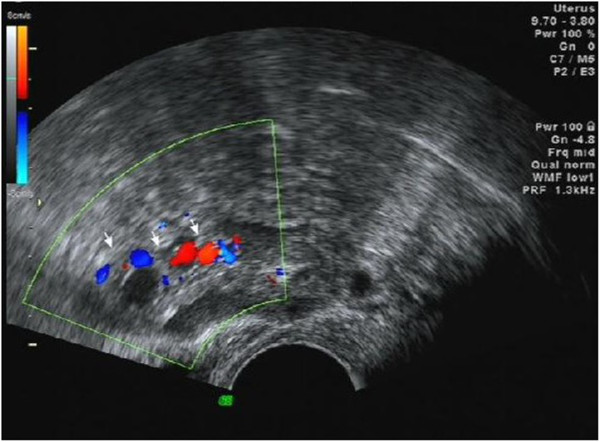
An ultrasound scan (on 16 December 2013) showed a 24mm×14mm left adnexal mass.

**Figure 4:**
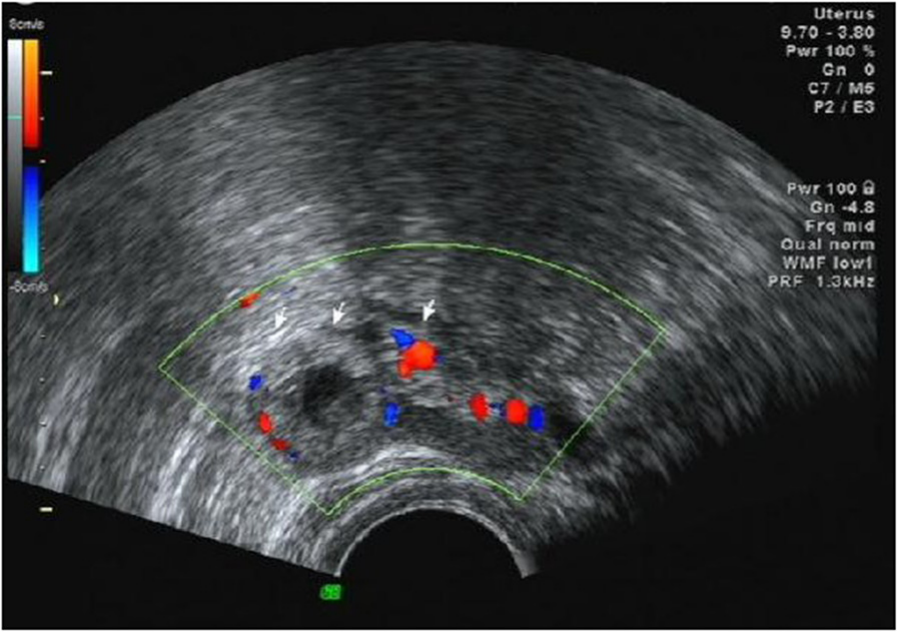
An ultrasound scan (on 18 December 2013) showed a 26mm×16mm left adnexal mass.

**Figure 5:**
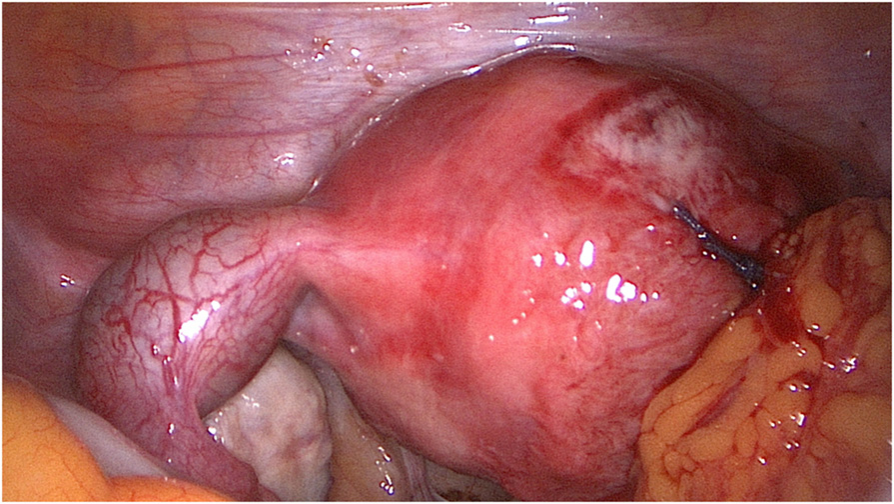
During the secondary surgery, a 30×20mm unruptured left ampullary pregnancy was found.

A 33-year-old Chinese woman presented to the gynecology clinic complaining of vaginal bleeding without abdominal pain. Her last menses was 42 days before the visit. She used no form of contraception. Our patient had no history of PID, no prior IUD, no use of fertility drugs and no pelvic surgery. Our patient had had conservative treatment in our hospital due to a left tubal pregnancy eight years before (a single-dose methotrexate (MTX) injection (50mg/m2) was administered, and she had an uneventful decrease in serum β-human chorionic gonadotropin (HCG) levels within two weeks) and one spontaneous abortion two years before. A physical examination revealed stable vital signs: blood pressure of 107/55mmHg and a pulse rate of 88/min. Her abdomen was soft and nontender. A vaginal examination revealed bloody discharge and a nontender pelvis. Her serum level of β-HCG was 6993.1IU/L and her progesterone level was 13.40μg/L. No gestational sac was detected in the uterine cavity by transvaginal ultrasound (TVUS) scan on 12 December 2013.The presumptive diagnosis of ectopic pregnancy was made and our patient was admitted to our gynecology ward. Upon admission, her serum level of β-HCG increased to 13721.3IU/L. A 45mm×29mm right adnexal mass was found by TVUS examination (on 13 December 2013) (Figure 1), and an ectopic pregnancy was suggested. Nothing abnormal was detected in the left adnexal area. The endometrial thickness was 10mm and the uterine cavity was empty. Having discussed the pros and cons of medical and surgical treatment options before the surgery, our patient and her family preferred the surgical treatment, and she insisted that she would like to preserve the integrity of the tube. After informed consent was obtained, a laparoscopy was performed, which revealed a 40×50mm nearly ruptured mass in the interstitial part of the right tube (Figure 2). A wedge resection of the right side of the uterine horn and partial (isthmus) resection of the right fallopian tube were done. An inspection of the left tube showed it was slightly thickened, which was considered to be a inflammatory sign because of the left tubal pregnancy eight years before. Pathology results confirmed placental villi in the right tube.Her serum level of β-HCG was 7373.5IU/L on the first day after surgery and increased to 10522.3IU/L the next day. On 16 December 2013, an ultrasound scan showed a 24mm×14mm anechogenic image in her left fallopian tube, mild thickened endometrium, and a small amount of fluid collection in the Douglas Pouch (Figure 3). Our patient had no obvious discomfort after the surgery so that the expectant therapy was executed.On 18 December 2013, her serum level of β-HCG rose to 13721.3IU/L and her progesterone level was 8.94μg/L. A 26mm×16mm left adnexal mass and a small amount of fluid was detected on TVUS examination. The endometrial thickness was 11mm and the uterine cavity was empty (Figure 4). Our patient was considered for BTP. A laparoscopy was performed after informed consent was obtained. A 30×20mm unruptured left ampullary ectopic pregnancy was found (Figure 5). A left linear salpingostomy and curettage were performed. Pathology results confirmed placental villi in the mass of the left tube and that there were no placental villi in the curettage specimen. Our patient was discharged home two days after surgery, in good condition. Her β-HCG serum level was 4128.0IU/L when she left hospital. She was followed up until her β-HCG serum level was normal.

## Discussion

An increase in the incidence of PID and increase in ARTs has caused a rise in BTP. BTP is the rarest form of ectopic pregnancy [4]. The incidence is reported to be five in one million deliveries [5]. Many cases are as a result of ARTs [6-9]. Nevertheless, it can be seen in normal natural menstrual cycles.

The mechanisms of BTP may include multiple ovulation, transperitoneal migration of trophoblastic tissue from one tube to another, or superfetation [10].

It is hard to diagnose BTP. The diagnosis is rarely confirmed before surgery. The clinical presentation of BTP is nonspecific. There are no unique clinical features to differentiate it from unilateral ectopic pregnancy. The diagnosis of BTP is hard to make based on serum levels of β-HCG, progesterone level and transvaginal ultrasound scan, particularly for nonsimultaneous bilateral tubal pregnancy. Sometimes, preoperative diagnosis made by imaging would cause misdiagnosis. To take our case as an example, the left ectopic pregnancy was neglected once the right pregnancy had been confirmed, which lead to our patient undergoing a secondary surgery. Most BTPs are diagnosed in the operating room. During the operation, once a gestational sac is found, a second gestational sac is not expected, and elevated serum levels of β-HCG are easily ignored. A lack of careful examination of the contralateral tube leads to misdiagnosis, just like in our case. Surgeons should always keep in mind the possibility of BTP, especially when facing a patient who has risk factors like PID, history of ectopic pregnancy and ART. Direct inspection of the contralateral tube in the operating room is the most effective method of diagnosing the second ectopic pregnancy. Laparoscopy is the gold standard for diagnosis of ectopic pregnancy, including BTP. The criteria for diagnosis of BTP were first suggested by Fishback [11] and later revised by Norris [12] who stated that microscopic demonstration of chorionic villi in each tube was sufficient for the diagnosis. A histopathological examination is essential to confirm the diagnosis, with the identification, at least, of chorionic villi in both tubes.

With regard to treatment for BTP, no guidelines are presently available on this topic. Laparoscopy is a diagnostic management and also a therapeutic process. The most proper and safest way to deal with BTP may be laparoscopic salpingostomy. But if both tubes are badly damaged or actively bleeding, bilateral salpingectomy may be a more suitable option [13]. For those infertile patients who received ART and were diagnosed with BTP, removal of both fallopian tubes might be a solution to exclude potential nonsimultaneous bilateral tubal pregnancy. Otherwise, careful examination of the contralateral tube during surgery and close follow-up of the β-HCG serum level after surgery facilitates discovery of potential nonsimultaneous bilateral tubal pregnancy for those patients who desire to preserve the other tube.

## Conclusions

This is a rarely seen case of spontaneous BTP. The incidence of unusual presentations of ectopic pregnancies has risen along with an increase in assisted reproductive techniques and pelvic inflammatory disease. A cautious examination of both fallopian tubes is necessary to avoid missing bilateral tubal pregnancy, especially for the patient who has risk factors like PID, history of ectopic pregnancy and ART. Making the diagnosis of BTP before or during surgery is critical so that the patient can avoid a secondary surgery. How to diagnose and treat BTP properly is a big responsibility for physicians. The purpose of this manuscript is to promote research into the epidemiology of BTP, improve diagnosis, and find the appropriate treatment.

### Consent

Written informed consent was obtained from the patient for publication of this case report and any accompanying images. A copy of the written consent is available for review by the Editor-in-Chief of this journal.

## Competing interests

The authors declare that they have no competing interests.

## Authors’ contributions

WL collected information and wrote the manuscript. GW analyzed and interpreted the patient data regarding diagnosis. TL and WS performed the surgery. All authors read and approved the final manuscript.

## References

[B1] Al-Quraan GA Al-Taani MI Nusair BM Arafat MR Khateeb MM Spontaneous ruptured and intact bilateral tubal ectopic pregnancy East Mediterr Health J 2007 13 972 974 17955781

[B2] Bettocchi S Nappi L Ceci O Vimercati A Selvaggi L Cormio G Vicino M Simultaneous bilateral tubal pregnancies and intrauterine pregnancy with five fetuses J Am Assoc Gynecol Laparosc 2004 11 195 196 10.1016/S1074-3804(05)60198-3 15200774

[B3] Shenoy JV Choudhary V Giles RW Bilateral ectopic pregnancy J Obstet Gynaecol 2005 25 612 613 10.1080/01443610500242689 16234159

[B4] Andrews J Farell S Spontaneous bilateral tubal pregnancies: a case report J Obstet Gynecol Can 2008 30 51 54 10.1016/S1701-2163(16)32713-X 18198068

[B5] Edelstein MC Morgan MA Bilateral simultaneous tubal pregnancy: case report and review of literature Obstet Gynecol Surv 1989 44 250 252 10.1097/00006254-198904000-00004 2652016

[B6] Musarrat J Babar A Jakimiuk J Bilateral ectopic pregnancy following ovulation induction JPM 2010 24 160 162

[B7] Tadeusz I Wojciech G Attur J Bilateral ectopic tubal pregnancy, following in vitro fertilization (IVF) Folia Histochem Cytobiol 2009 47 147 148 10.2478/v10042-009-0093-0 20067887

[B8] Burgos-San Cristobal DJ Agirregoikoa JA Albisu M Simultaneous bilateral ectopic pregnancy after IUI Rev Iberoam Fertile Reprod Hum 2004 21 349 353

[B9] Ghaffari F Eftekhari Yazdi P Kiani K A case report of bilateral tubal ectopic pregnancy following day 5 embryo transfer Arch Med Sci 2011 7 1087 1088 22328897 10.5114/aoms.2011.26626 PMC3265006

[B10] Tabachnikoff RM Dada MO Woods RJ Rohere D Myers CP Bilateral tubal pregnancy: a report of an unusual case J Reprod Med 1998 43 707 709 9749426

[B11] Fishback HR Bilateral simultaneous tubal pregnancy Am J Obstet Gynecol 1939 37 1035 1037

[B12] Pezzani M A case of simultaneous interstitial bilateral pregnancy Acta Gent Med Gemellol 1976 35 325 327 10.1017/s0001566000014367 1031536

[B13] Ataíde Lobo R Patrício L Milheiras E Cordeiro A Hermida M Bilateral tubal Ectopic PregancyGravidez ectópica t ubária bilateral Acta Obstet Ginecol Port 2012 6 141 144

